# Enterovirus-Rhinovirus-Induced Acute Respiratory Distress Syndrome in Adults: A Case Report and Short Literature Review

**DOI:** 10.1155/2023/8887955

**Published:** 2023-11-03

**Authors:** Eirini Avgoustou, Aikaterini Spyridaki, Giorgos Pothitos, Antonios Papadopoulos, Spyridon Kois, Foula Vassilara

**Affiliations:** ^1^5^th^ Department of Internal Medicine, Hygeia Hospital, Athens, Greece; ^2^Department of Internal Medicine, Sismanoglio-Amalia Fleming General Hospital, Athens, Greece; ^3^4^th^ Department of Internal Medicine, Medical School, National and Kapodistrian University of Athens, Athens, Greece

## Abstract

Enteroviruses and rhinoviruses (EV-RV) are small RNA viruses that usually cause the common cold and asthma exacerbations. Although EV-RV-induced acute respiratory distress syndrome (ARDS) is common in children, only scattered reports of ARDS in adults have been published. The diagnosis has been greatly facilitated by the advent of molecular techniques, namely, real-time polymerase chain reaction (RT-PCR). EV-RV can cause ARDS by stimulating a cytokine cascade. No antiviral therapy has yet been approved, and treatment is entirely supportive. Herein, we report a rare case of EV-RV infection in an afebrile adult with dyspnea that rapidly progressed to acute lung injury and ARDS. EV-RV was isolated with multiple real-time PCR in nasopharyngeal and bronchial specimens, while no other pathogen was detected. We also present an up-to-date review of relevant literature, in an attempt to stress the importance of the early identification of viral culprits, which can minimize the use of invasive diagnostic procedures and antibiotic agents.

## 1. Introduction

Rhinoviruses (RVs), first isolated in 1956, are the leading causes of upper respiratory tract infections worldwide. They are small, nonenveloped, positive-sense, single-stranded ribonucleic acid (ssRNA) viruses of approximately 7,200 bp. RVs are an extremely heterogeneous group of viruses belonging to the *Enterovirus* genus within the Picornaviridae family [1]. To date, over 165 RV genotypes have been described. With the use of advanced molecular techniques, three distinct RV groups designated as rhinovirus A (RV-A), rhinovirus B (RV-B), and rhinovirus C (RV-C) (International Committee on Taxonomy of Viruses, ICTV) have been isolated, with marked phylogenic diversity such that immunity to any subtype is unlikely to confer protection to the others [2]. Typically associated with the common cold, accounting for approximately 50% of cases, they are nowadays known to be also associated in some cases with severe and potentially fatal conditions such as aseptic meningitis, encephalitis, myocarditis, viral pneumonia, and ARDS [3–5]. The substantial advancement in the understanding of their clinical spectrum is mainly due to the development of molecular methods that have facilitated the detection of these viruses. To date, there is no generally approved antiviral agent for the treatment of RV infection, which remains mainly supportive. The development of a vaccine effective for its prevention has also failed so far.

## 2. Case Report

An 89-year-old male presented to the emergency department complaining of dyspnea that started four days ago and was gradually worsening. His past medical history included type 2 diabetes mellitus, dyslipidemia, arterial hypertension, and atrial fibrillation.

**Figure 1:**
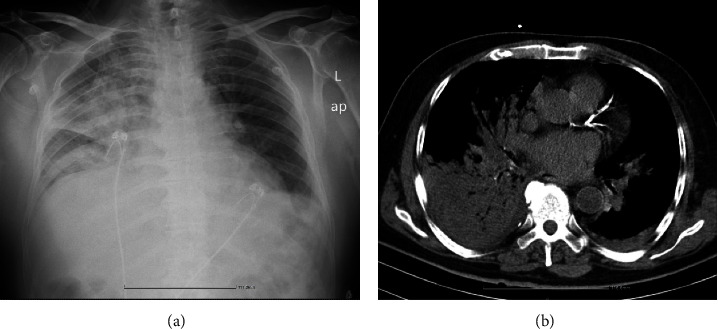
(a) Chest X-ray (bedside) and (b) chest CT scan on admission.

**Figure 2:**
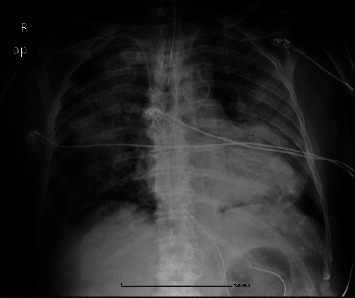
Chest X-ray (bedside) on the fourth day of hospitalization in the ICU after clinical deterioration depicting diffuse bilateral opacities.

On physical examination, the patient was ill-appearing, tachypnoic (40 breaths per minute) and hypoxic (oxygen saturation 77% on ambient air). His blood pressure was 120/80 mmHg and his heart rate was 85 bpm with irregular rhythm. Bilateral crackles were heard on auscultation. Arterial blood gas parameters indicated hypoxic respiratory failure with PaO_2_ 62.4 mmHg (Venturi mask 60%) and lactate 2.9 mmol/L (PaO_2_/FiO_2_ ratio = 105). Complete blood count analysis displayed leucocytosis (white blood cells 13.600/*μ*L) with left shift, and biochemistry showed elevated troponin levels and mildly elevated B-type natriuretic peptide (BNP) and creatine phosphokinase (CPK). Computed tomography of the chest showed multiple airspace consolidations, predominately in the right lung and to a lesser degree in the left lung and the glottis, mainly with central distribution (Figure 1). A bedside transthoracic echocardiogram revealed ejection fraction 45% and mild regurgitation of the aortic and mitral valves. The patient was admitted to the intensive care unit (ICU). The empiric broad-spectrum antimicrobial treatment (ceftriaxone and levofloxacin) was administered intravenously immediately after drawing blood cultures, and bronchodilators were initiated. On the second day of hospitalization, high-dose intravenous steroids were started as adjuvant therapy (methylprednisolone 40 mg every 8 hours), due to the rapid decline (Figure 2), and he received oxygen support through a high-flow nasal cannula with flow rates of up to 60 liters/min. Nasopharyngeal specimens were collected twice, and multiplex real-time polymerase chain reaction (RT-PCR) was applied (BioFire® FilmArray Respiratory Panel *plus*, bioMérieux S.A., Marcy-l'Etoile, France). The results were positive only for *Rhinovirus/Enterovirus* isolation; all other respiratory pathogens were negative (*adenovirus*, *coronavirus HKU1*, *NL63*, *229E*, and *OC4*, *human metapneumovirus*, *influenza A and B viruses*, *human parainfluenza 1*, *2*, *3*, *and 4 viruses*, *respiratory syncytial virus*, *Bordetella Pertussis*, *Chlamydophila pneumoniae*, and *Mycoplasma pneumoniae*). Urine antigen tests for *Streptococcus pneumoniae* and *Legionella pneumophila* were negative.

The patient was continuously worsening with decreasing diuresis on the third day and aerometric deterioration on the fourth day, requiring endotracheal intubation and mechanical ventilation. A bedside chest X-ray showed bilateral diffuse opacities, compatible with ARDS (Figure 2). A new multiplex RT-PCR assay on bronchial washing fluid detected again nothing but *Rhinovirus/Enterovirus*. We used the upper respiratory panel, since the molecular panel for the lower respiratory tract was not developed at that time (2018). The bacterial cultures of blood, urine, and tracheal secretions remained sterile, establishing the diagnosis of EV-RV-induced ARDS. The patient was in shock and multiorgan failure despite titration of vasopressors. Antimicrobials were escalated to meropenem, linezolid, and fluconazole, but the outcome was unfavorable, and the patient passed away.

## 3. Discussion

In recent years, other respiratory viral pathogens have been overshadowed by coronavirus disease 2019 (COVID-19). *Rhinoviruses* are among the most common causes of human infections, with a reported peak in incidence in the late spring and early fall [1, 6]. Recently, the relative importance of *Rhinovirus* as an important pathogen has been reconsidered with the advent of more sensitive detection methods such as RT-PCR for multiple pathogens, since it is increasingly being detected in critically ill patients. Indeed, RV is present in 25–30% of severe community-acquired pneumonias (CAPs) [7]. The exact pathogenetic mechanism of RV infection has not yet been fully elucidated; however, there may be a combination of both direct viral-mediated injury of respiratory epithelial cells and a cytokine-induced dysregulated innate host inflammatory response (including stimulation of IFN-*β*, IFN-*γ*, IL-1, IL-6, and IL-8 expression mediated in part by a NF-k*β*-dependent transcriptional activation pathway) [1]. Kinins (i.e., bradykinin) may also play a role in the mechanism of the symptomatic disease, as suggested by their elevated levels in nasal lavage fluid specimens of symptomatic subjects, when compared to healthy controls [1]. This cytokine cascade increases the permeability of the alveolar-capillary membrane, resulting in injury of the lung parenchyma, termed diffuse alveolar damage, and therefore in hypoxia, pulmonary edema, plasma protein leakage, and further macrophage and neutrophil infiltration [8]. Hyaline membranes in the alveoli can be observed on histological examination. During this inflammatory process, alterations in the composition and functionality of the surfactant lead to alveolar collapse [8]. RV infection triggers not only cytotoxic but also humoral immune responses, with the development of serotype-specific neutralizing serum antibodies. Unfortunately, the existence of more than 160 different known RV serotypes and the fact that there is little cross-neutralization among serotypes explain the failure of developing an effective vaccine at present [1, 6].

### 3.1. Clinical Presentation

EV and RV mainly cause infections in the pediatric population. In adults, the clinical spectrum varies widely, from asymptomatic infection, cough and nasal congestion, wheezing, and dyspnea, to fulminant respiratory failure and fatal ARDS [9]. In children, they have been associated with more severe disease than other common viruses (*RSV, influenza A/B,* and *parainfluenza 1–3*), especially with underlying cardiorespiratory or immunodeficient/metabolic conditions, and have been proved to cause otitis media, sinusitis, bronchiolitis, exacerbations of asthma, and cystic fibrosis [5, 10–12]. In immunocompetent adults, rhinovirus most commonly causes a self-limited influenza-like illness and may be responsible for more than 50% of common colds during the fall and spring. In this population, rare cases of severe EV-RV disease have been reported [4, 8, 9, 13–15]. The development of the aforementioned molecular methods has greatly facilitated the recognition of *Rhinovirus* as a significant cause of severe acute lower respiratory tract infections. Immunocompromised hosts, including those with diabetes mellitus, human immunodeficiency virus (HIV) infection, hematologic malignancies, or organ transplantation, are particularly prone to severe *Rhinovirus* infection [1, 6, 16]. Viral pneumonia should also be considered in immunocompetent patients when they are elderly or with severe comorbidities (especially structural lung disease or chronic kidney disease) [17]. Indeed, unusually high morbidity and mortality rates have been described in *Rhinovirus* outbreaks among elderly residents in long-term care facilities [18]. Smoking is another important independent risk factor: smokers with pneumonia are about three times more likely to be admitted to an ICU than nonsmokers [4]. Several studies have also demonstrated a significant association between RV infection and exacerbations of asthma or chronic obstructive pulmonary disease [1, 6]. Another important consideration is that RV may predispose to bacterial superinfections (i.e., *Streptococcus pneumoniae* and *Staphylococcus aureus*), as well as fungal pathogens (usually *Aspergillus* species in immunocompromised hosts), resulting in high morbidity and mortality rates [1, 5, 16].

EV-RV can cause ARDS. Its diagnosis is based on the Berlin criteria in which the development of respiratory distress should occur within one week of a respiratory viral infection, with bilateral opacities on chest radiography and minimal to absent contribution of cardiogenic pulmonary edema or volume overload states [19]. ARDS is characterized by diffuse inflammation of the lungs, leading to severe respiratory distress and hypoxemia refractory to oxygen therapy, and ultimately respiratory failure, in which case endotracheal intubation and mechanical ventilation are warranted. EV-RV-induced ARDS is common in the pediatric population, especially in children with a history of asthma; in adults, however, only a few cases of EV-RV-associated ARDS have been reported so far in the literature [8, 9, 13–15] (Table 1). ARDS is associated with high mortality rates, ranging from 26% to 58% [13].

Notably, the two reported cases that, despite all medical efforts eventually died, share some important characteristics as follows: they both were elderly people, with severe comorbidities, who also presented with a significant delay since the onset of their symptomatology.

### 3.2. Diagnostic Evaluation

The most common diagnostic technique for EV-RV is RT-PCR. Specimens should be collected for laboratory analysis as soon as possible after the onset of symptoms, since RV titers are highest during the first two days of presentation [1]. For the diagnosis of an upper respiratory tract infection, nasopharyngeal swabs are used, while for the lower tract, samples include tracheal or bronchial aspirate, BAL fluid, or, less commonly, lung biopsy. In our case, we were able to collect specimens from both the upper and lower respiratory tracts, establishing a firm diagnosis of EV-RV-induced ARDS, taking into account that no other pathogen was isolated with the most sensitive diagnostic methods. The use of upper respiratory tract samples alone for the diagnosis of CAP is controversial since nasopharyngeal samples are not generally considered adequate for the diagnosis [20]. In the study by Hong et al. [17], viruses were identified in the bronchoalveolar lavage (BAL) fluid from nearly two-thirds of the subject patients (62.7%), but only in 37.3% of their nasopharyngeal specimens.

Multiple real-time PCR techniques have been developed since the late 1980s [1]. PCR-based assays targeting respiratory viruses and atypical bacteria have deepened our understanding of CAP etiology. The improved detection of viruses indicates that CAP must no longer be considered as exclusively bacterial and may obviate unnecessary antimicrobial use. Recently, rapid PCR panels for viruses and bacteria involved in CAP have been developed that enable rapid detection of pathogens and are a useful tool in severe respiratory tract infections, where time is critical for the management and the outcome of the patient [21]. These panels have been evaluated by several studies, which confirm their high accuracy and efficacy to detect pathogens when compared to standard diagnostic methods (culture of the respiratory tract and blood samples, urine antigen tests for *Pneumococcus* and *Legionella*, etc.) [22]. Notably, while quantitative testing sounds attractive for the prediction of clinical progression, several factors such as the sample type, collection procedure, age, and immune status of the patient affect viral load and pose a challenge in any attempt to provide an accurate and reliable quantification and its clinical interpretation [1].

Serology is only useful for epidemiologic purposes since antibodies begin to rise from 1 to 3 weeks postinfection, making them useless in the acute clinical setting [1]. Similarly, conventional virus cultures, while important for studies of virus characteristics and disease pathogenesis, have to be inoculated for up to 14 days and are rarely used in clinical practice [1].

### 3.3. Treatment

There is no established treatment strategy for rhinoviral pneumonia. Over the years, several agents have been evaluated as follows: capsid-binding agents (pleconaril, vapendavir, and pirodavir), proteolytic enzyme inhibitors (rupintrivir), alpha-2-interferon, ribavirin, Echinacea preparations, zinc, and antihistamines [1]. Some of them have shown a trend towards reduced symptom severity and duration, but none have been approved by the U.S. Food and Drug Administration due to either nonstatistically important clinical benefits or serious side effects. Therefore, RV treatment remains to date entirely supportive.

The treatment of RV-induced ARDS is no different from that of any other cause, with correction of hypoxemia, permissive hypercapnia, and prone positioning [8, 9, 15]. Corticosteroids have been considered as a potentially effective therapy for ARDS since the syndrome's original description in 1967 [23–25]. Despite the numerous relevant clinical trials over the past several decades, the topic remains controversial since results have been conflicting. Corticosteroids might be harmful in some viral pneumonias, such as influenza pneumonia, in which steroids have been reported to delay viral clearance; in contrast, in COVID-19 disease, dexamethasone has been proved to increase survival [23]. These data emphasize the importance of identifying the specific cause of ARDS. To our knowledge, there is no study establishing corticosteroids as an effective treatment option for RV pneumonia, and further trials need to be undertaken. For patients who require invasive mechanical ventilation, a low-tidal-volume strategy is recommended (4–6 mL/kg of ideal body weight) to prevent barotrauma [8, 9, 23, 26]. Severe ARDS can be additionally managed using neuromuscular blockade during the first 48 hours following intubation [8].

### 3.4. Prevention

RV person-to-person transmission can be airborne (small and large aerosol particles) or via contact (either direct or with fomites) [1]. Therefore, prevention strategies should stress the importance of social distancing, use of respiratory masks, and hand hygiene. Furthermore, to date, all efforts to develop an effective vaccine have been unsuccessful (see above).

## 4. Conclusion

With the increasing use of multiplex molecular assays currently available in most microbiology facilities, respiratory virus detection has entered a new era. As data accumulate to prove the high incidence of *Enterovirus-Rhinovirus*, there has been increasing awareness of their widespread and sometimes serious presentation, making them more and more commonly identifiable as causative agents in severe lower respiratory tract infections in both adult and children populations, immunocompromised or not. Since there are still very few reports of EV-RV-induced ARDS, we hope to have demonstrated the need for the medical community to regard EV-RV as pathogens responsible for much more than a common cold. We aim to stress out the importance of taking rhinoviruses into account in the differential diagnosis of pneumonia causing severe ARDS in patients regardless of age. Early identification of EV-RV infection using multiplex molecular assays could potentially reduce unnecessary invasive diagnostic procedures and use of antibiotics.

## Figures and Tables

**Table 1:** Comparison of published cases of ER-RV-induced ARDS in adults.

Case references	Study types	Age (years)/sex	Symptom on presentation	Radiographic findings	Method for diagnosis	Treatment	Aerometric support	Outcome
Present case	Case report	89/M	Dyspnea for 4 days	Diffuse bilateral opacities	Multiplex PCR of nasopharyngeal swab and bronchial washing fluid	Ceftriaxone, levofloxacin ⟶ meropenem, linezolid, fluconazole, methylprednisolone 40 mg × 3	Endotracheal intubation/mechanic ventilation	Death
Ngu et al. [8]	Case report	59/F	Respiratory distress, dry cough for 1 week	Diffuse bilateral multifocal airspace opacities	Multiplex PCR of nasopharyngeal swab	Vancomycin, levofloxacin, piperacillin/tazobactam, high-dose IV steroids	High-flow nasal cannula	Recovery
Cecchini et al. [9]	Case report	67/M	Fatigue, lightheadness ⟶ rapid increase in oxygen needs	Extensive bilateral ground-glass opacities	Multiplex PCR	Vancomycin, cefepime, dexamethasone 20 mg × 1	Endotracheal intubation/mechanic ventilation	Recovery
Soni et al. [13]	Case report	22/F	Respiratory distress, productive cough, wheezing for 1 week	Pulmonary vascular congestion, left lower lobe infiltrate	Multiplex PCR of nasopharyngeal swab	Azithromycin, ceftriaxone	Endotracheal intubation/mechanic ventilation	Recovery
Ayala et al. [14]	Case report	60/F	Fever, respiratory distress	Bilateral pulmonary ground-glass opacities	Multiplex PCR of nasopharyngeal swab	Vancomycin, piperacillin/tazobactam ⟶ meropenem	Endotracheal intubation/mechanic ventilation	Recovery
Hamid et al. [15]	Case report	70/F	Shortness of breath for 8 days	Multifocal bilateral atypical pneumonia	Multiplex PCR of nasopharyngeal swab	Empiric antibiotic treatment, methylprednisolone 40 mg × 2 ⟶ 1 g × 3, dapsone 100 mg × 1	Endotracheal intubation/mechanic ventilation	Death

## Data Availability

All the necessary data and information are within the article.
